# Projecting the spatiotemporal carbon dynamics of the Greater Yellowstone Ecosystem from 2006 to 2050

**DOI:** 10.1186/s13021-015-0017-6

**Published:** 2015-03-19

**Authors:** Shengli Huang, Shuguang Liu, Jinxun Liu, Devendra Dahal, Claudia Young, Brian Davis, Terry L Sohl, Todd J Hawbaker, Ben Sleeter, Zhiliang Zhu

**Affiliations:** 1 ASRC Federal InuTeq, Contractor to the U.S. Geological Survey (USGS) Earth Resources Observation and Science (EROS) Center, 47914 252nd Street, Sioux Falls, SD 57198 USA; 2 USGS EROS Center, 47914 252nd Street, Sioux Falls, SD 57198 USA; 3 Contractor to USGS Western Geographic Science Center, 345 Middlefield Rd, Menlo Park, CA 94025 USA; 4 Stinger Ghaffarian Technologies (SGT), Inc., Contractor to the USGS EROS Center, Sioux Falls, SD 57198 USA; 5 Innovate!, Inc. Contractor to the USGS EROS Center, Sioux Falls, SD 57198 USA; 6 U.S. Geological Survey, Denver, CO USA; 7 U.S. Geological Survey, Reston, VA USA

**Keywords:** Climate change, Wildfires, Land cover and land use, Carbon sequestration, Yellowstone

## Abstract

**Background:**

Climate change and the concurrent change in wildfire events and land use comprehensively affect carbon dynamics in both spatial and temporal dimensions. The purpose of this study was to project the spatial and temporal aspects of carbon storage in the Greater Yellowstone Ecosystem (GYE) under these changes from 2006 to 2050. We selected three emission scenarios and produced simulations with the CENTURY model using three General Circulation Models (GCMs) for each scenario. We also incorporated projected land use change and fire occurrence into the carbon accounting.

**Results:**

The three GCMs showed increases in maximum and minimum temperature, but precipitation projections varied among GCMs. Total ecosystem carbon increased steadily from 7,942 gC/m^2^ in 2006 to 10,234 gC/m^2^ in 2050 with an annual rate increase of 53 gC/m^2^/year. About 56.6% and 27% of the increasing rate was attributed to total live carbon and total soil carbon, respectively. Net Primary Production (NPP) increased slightly from 260 gC/m^2^/year in 2006 to 310 gC/m^2^/year in 2050 with an annual rate increase of 1.22 gC/m^2^/year. Forest clear-cutting and fires resulted in direct carbon removal; however, the rate was low at 2.44 gC/m^2^/year during 2006–2050. The area of clear-cutting and wildfires in the GYE would account for 10.87% of total forested area during 2006–2050, but the predictive simulations demonstrated different spatial distributions in national forests and national parks.

**Conclusions:**

The GYE is a carbon sink during 2006–2050. The capability of vegetation is almost double that of soil in terms of sequestering extra carbon. Clear-cutting and wildfires in GYE will affect 10.87% of total forested area, but direct carbon removal from clear-cutting and fires is 109.6 gC/m^2^, which accounts for only 1.2% of the mean ecosystem carbon level of 9,056 gC/m^2^, and thus is not significant.

## Background

Climate change affects ecosystem carbon dynamics through multiple pathways, including altering biogeochemical cycles (e.g., productivity), disturbance regimes (e.g., fire), and land use [1,2]. For example, climate impacts land use by influencing suitability of the landscape to support a given land use. These climate change effects are often region specific and show spatial variability [3]. The study of carbon dynamics under climate change is always challenging because of the combined influence of fire regimes, land use change, data uncertainties, and spatial heterogeneity.

Fire is a major ecosystem disturbance and more frequent and severe fires caused by warmer, drier conditions under climate change might reduce forest productivity and carbon storage [4]. In many coniferous forests, stand-replacing fires affect carbon cycling and storage over large spatial extents and long time periods [5], and increasing fire frequency with climate change has short-term and long-term effects for carbon storage [5]. For example, Westerling, et al. [6] used climate projections and examined the likely changes in occurrence, size, and spatial location of large fires (>200 hectares) in the Greater Yellowstone Ecosystem (GYE). They found continued warming could completely transform GYE fire regimes by the mid-21st Century, with profound consequences for many species and for ecosystem processes, including carbon storage [6]. Therefore, quantifying changes in forest carbon after disturbances is essential for managing future carbon emissions, especially given the uncertainties about forest carbon storage under future climate scenarios [7].

Changes in land use, including forest harvesting, continue in many landscapes, and those changes cannot only impact climate directly through alterations in the surface-energy budget [8] but also affect carbon sequestration [4]. Land use can be projected from climate change scenarios using approaches such as IMAGE [9]. The resulting land use changes influence carbon dynamics, which is demonstrated by Karjalainen, et al. [10], who compared a management-as-usual scenario with a multifunctional management scenario and evaluated the carbon accounting using the European Forest Information Scenario Model (EFISCEN). However, forecasting future trends in land use or forest management and examining the impact on carbon remains difficult.

Many General Circulation Models (GCMs) are used to predict climate change for given emission scenarios. However, predictions vary among GCMs, which may have a major effect on carbon modeling [4]. For example, Schaphoff, et al. [11] used five GCMs from one scenario and found the increase in global Net Primary Production (NPP) ranged from 16% to 32%. Therefore, multiple GCM outputs are desired to simulate the carbon differences among different GCMs.

The spatial heterogeneity of data adds more complexity to carbon modeling under climate change for several reasons. First, the trends of climate change are non-uniform through space and time; therefore, the impact on carbon cycling shows heterogeneity [1,2]. Second, fire regimes may show geographic differences because of the spatial variation in precipitation [12]. Third, landscape patterns in forest structure and stand age need to be considered in estimates of future carbon flux across landscapes [13], which is confirmed by Smithwick, et al. [14], who quantified the carbon storage for young and mature stands and showed the variation in tree density might influence carbon flux under differing climate change scenarios. By further incorporating climate change, Smithwick, et al. [15] integrated CENTURY version 4.5 to project future carbon stocks of individual stands under different climate scenarios and fire regimes based on three GCMs, Community Climate System Model (CCSM) 3.0, Centre National de Recherches Météorologiques Circulation Model (CNRM) CM 3.0, and Geophysical Fluid Dynamics Laboratory Climate Model (GFDL CM) 2.1, forced with the A2 emissions pathway. They reaffirmed spatial variation is critical for understanding the spatial pattern in total ecosystem carbon stocks across the landscape and ignoring the spatial variation across heterogeneous landscapes may lead to erroneous expectations on ecosystem carbon storage.

A flexible modeling approach that can incorporate sufficient interaction, contingency, and site specificity is required to examine how concurrent changes in climate, disturbance regimes, and land use influence ecosystem carbon budgets [16,17]. The GYE is a nearly intact ecosystem subject to the changes in climate, land use, and wildfire disturbance. How these concurrent changes influence the carbon dynamics at the landscape level remains unclear. The objective of this study was to use multi-source GCMs to model the spatiotemporal carbon storage in GYE associated with changes in fire, land use, and climate. We asked to what degree climate change would affect the carbon pools and fluxes in this ecosystem and whether GYE would be a carbon sink or carbon source. We hypothesized that carbon dynamics under climate change would show significant spatial variation due to the highly heterogeneous landscape in GYE. We additionally hypothesized that economy development and climate warming would result in more forest disturbance of clear-cutting and wildfires. To achieve the goal, we selected three climate change scenarios (A2, A1B, B1), then processed the CENTURY model under the General Ensemble Biogeochemical Modeling System (GEMS; [18]) using three GCMs for each scenario. We also projected the land use change and fire occurrence and incorporated their projections into the carbon accounting. By incorporating all these components, we examined the spatial and temporal carbon change of the GYE under climate change during 2006 to 2050.

## Methods

### Study area

**Figure 1:**
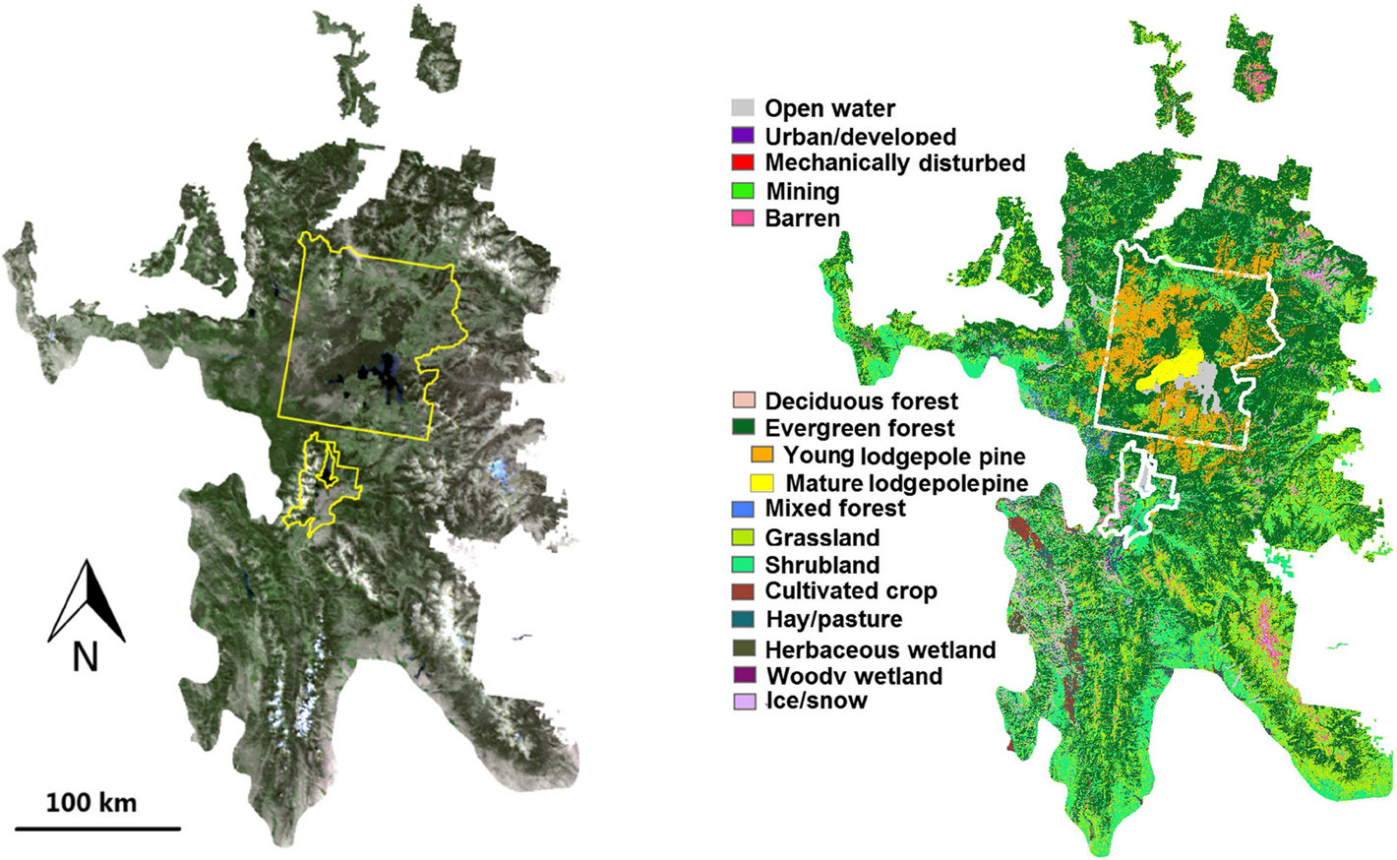
**Study area of Greater Yellowstone Ecosystem (GYE).** The left is a Moderate Resolution Imaging Spectroradiometer (MODIS) image composite (showing band 1 as red, band 4 as green, and band 3 as blue) from July 21, 2006. Dark green indicates unburned forest, white indicates high-mountain bare rock or glacier, dark blue indicates water bodies, and light green indicates regenerating forest or non-forested area. The yellows polygons are national parks. On the right is 2006 land cover, where young lodgepole pine is an evergreen area burned in 1988 and mature lodgepole pine is a large unburned evergreen forest.

GYE is one of the last remaining large, nearly intact temperate ecosystems in North America. GYE comprises 80,000-km^2^ of the Rocky Mountains and encompasses two national parks, six national forests, and three wildlife refuges (Figure 1). The GYE features a continental climate of cold, snowy winters and warm, dry summers. The mean high temperatures in July are 21°C on the plateau and 24°C at mid-elevations. The mean low temperature in January is about −15°C across the region. The mean annual precipitation ranges from 600–1100 mm on the plateau to 350–650 mm at mid-elevations [19]. Lodgepole pine (*Pinus contorta*), which occupies infertile volcanic (rhyolitic) soils across the Yellowstone Plateau, and Douglas fir (*P. menziesii*), which occupies moderately fertile (non-rhyolitic, sedimentary) soils on adjacent sloping terrain, account for about two-thirds of the forested area of the GYE. Other tree species include Engelmann spruce (*Picea engelmannii*), subalpine fir (*Abies lasiocarpa*), and whitebark pine (*Pinus albicaulis*) on moist/high-elevation sites, or limber pine (*Pinus flexilis*) and Rocky Mountain juniper (*Juniperus scopulorum*) on dry/low-elevation sites [19].

Wildland fires are historically common in the GYE. The fire return intervals in GYE forests have been about 100–300 years for the past 10,000 years, and the intervals in the lower elevation forest-steppe vegetation is about 75–100 years [6]. An extensive fire event burned the area in 1988, which resulted in heterogeneous lodgepole pine regeneration [20]. Fine litter, branches, and foliage can be consumed and live trees can be killed during canopy fires, but the carbon lost from the pools of tree boles, downed wood, and soil is low [21]. A period of 70–100 years is required to recover the carbon losses following lodgepole pine stand-replacing fire [22].

A climate change study has indicated GYE will have elevated temperatures, reduced winter precipitation, earlier snowmelt and spring runoff, and higher potential evapotranspiration in the future [23]. Under these changes, the number of large fires has increased in the past 25 years, and this trend is expected to continue with global warming [24]. The fire rotation may decrease to <30 years with a 4.5–5.5°C warmer spring-summer temperature by mid-century [6]. Although largely undeveloped, GYE is also undergoing a transition in human demographics and economics. The population increased 58% and the area of rural lands increased 350% from 1970 to 1999 [25]. With local economic development, the communities of the GYE have undergone rapid change, especially within the 32% of the GYE that is privately owned [25]. For example, during 1975–1995, there were increases in burned and urban areas but decreases in conifer habitats [26]. Forest harvesting in the national forests during the mid-20th Century created patchy mosaics of small, dispersed clear-cuts in some areas, but extensive portions of the GYE remain federally protected wildlands [27].

### Datasets

**Figure 2:**
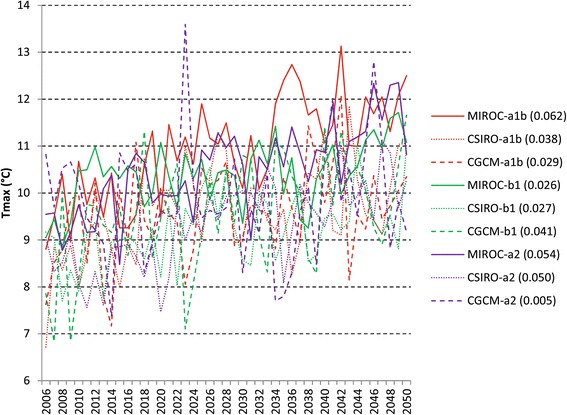
**Projected annual average maximum temperature (T**_**max**_ **) for different data-scenario combinations.** The values in the legend that are in parentheses are the slopes of linear regressions, indicating the annual increasing rates.

**Figure 3:**
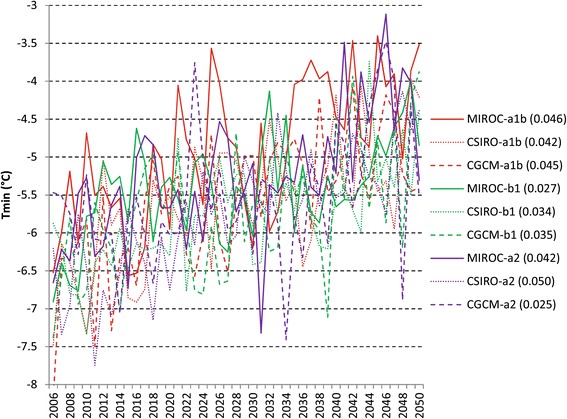
**Projected annual average minimum temperature (T**_**min**_ **) for different data-scenario combinations.** The values in the legend that are in parentheses are the slopes of linear regressions, indicating the annual increasing rates.

**Figure 4:**
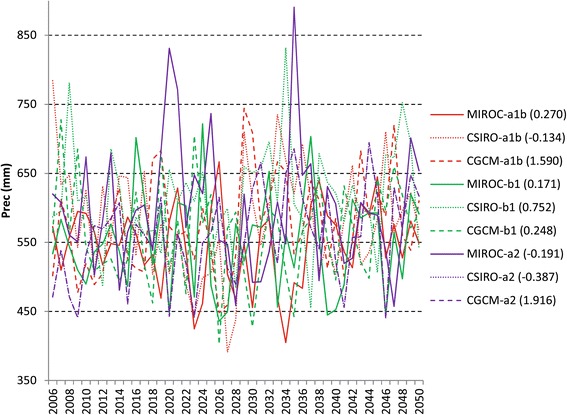
**Projected annual average precipitation (Prec) for different data-scenario combinations.** The values in the legend that are in parentheses are the slopes of linear regressions, indicating the annual increasing (+) or decreasing (−) rates.

The Intergovernmental Panel on Climate Change Special Report on Emission Scenarios (IPCC-SRES) published different scenarios exploring future emissions pathways [28]. Three IPCC-SRES scenarios (A1B, A2, and B1) were used in this study. For each scenario, we collected climate data from the Coupled General Circulation Model 3.1 (CGCM 3.1) [29], Australia’s Commonwealth Scientific and Industrial Research Organization Mark 3.0 model (CSIRO–Mk3.0) [30], and the Model for Interdisciplinary Research on Climate version 3.2, medium resolution (MIROC 3.2-medres) [31]. Based on these three scenarios and three GCM models, nine data-scenario combinations were used: CGCM-A1B, CGCM-A2, CGCM-B1, CSIRO-A1B, CSIRO-A2, CSIRO-B1, MIROC-A1B, MIROC-A2, and MIROC-B1. In GYE, all climate projections show an obviously increasing trend in maximum and minimum temperatures (Figures 2 and 3), but this uniform trend is not observed for projected precipitation (Figure 4): the largest precipitation increase was from CGCM-A2 with an increasing rate of 1.9 mm/y followed by CGCM-A1B (1.6 mm/y) and CSIRO-B1 (0.8 mm/y); the largest precipitation decrease was from CSIRO-A2 with a decreasing rate of 0.39 mm/y followed by MIROC-A2 (0.2 mm/y) and CSIRO-A1B (0.1 mm/y).

Topography (elevation, slope, and aspect) data were retrieved from the U.S. Geological Survey (USGS) National Elevation Dataset [32]. Forest data were collected from U.S. Forest Service’s Forest Inventory & Analysis [33]. Soil data were mainly compiled from the Soil Survey Geographic (SSURGO) database [34], but the State Soil Geographic (STATSGO) database [35] was used where SSURGO data were not available.

### Projections and modeling

We used the GEMS-CENTURY model to simulate the carbon dynamics in GYE by incorporating wildfire and land use change. The land-cover and land use projections, wildfire projections, and biogeochemical modeling are described in the following sections. The models used in this local study were validated for use at regional and national scale [36]; therefore, this study is a “bird’s-eye view” of the GYE and does not take into account some specific features of this ecosystem.

#### Land Use and Land-Cover (LULC) projection from 2006 to 2050

IPCC-SRES storylines were designed to represent different socioeconomic development pathways, with the assumption of different driving forces such as energy sector, population growth, technological innovation, economic growth, environmental protection, and regional/global orientation [28] (Table 1). A scenario downscaling process was used to translate the coarse-scale scenario data to finer geographic scales while maintaining consistency with the original dataset and local data [37]. A global integrated assessment model (IAM) was then used to supply future projections of land use at the national scale. An accounting model was developed to refine the national-scale IAM projections and to downscale to the ecoregion where the study area is located [38]. The spatially explicit land-cover projections from 2006 to 2050 were developed using a spatially explicit LULC change model called the FOREcasting SCEnarios of land use change (FORE–SCE) model [39]. The FORE–SCE model used separate but linked “Demand” and “Spatial Allocation” components to produce spatially explicit, annual LULC maps. The “Demand” component provided aggregate-level quantities of LULC change for a region, or a “prescription” for the overall regional LULC proportions. The “Spatial Allocation” component ingested “Demand” and produced spatially explicit LULC maps using a patch-based allocation procedure [39].

**Table 1:** **Assumptions about the primary driving forces affecting land-use and land-cover change**

**Driving forces**	**A1B**	**A2**	**B1**
Population growth (global and United States)*	Medium. Globally, 8.7 billion by 2050, then declining; in the United States, 385 million by 2050	High. Globally, 15.1 billion by 2100; in the United States, 417 million by 2050	Medium. Globally, 8.7 billion by 2050, then declining; in the United States, 385 million by 2050
Economic growth*	Very high. U.S. per-capita income $72,531 by 2050	Medium. U.S. per-capita income $47,766 by 2050	High. U.S. per-capita income $59,880 by 2050
Regional or global orientation	Global	Regional	Global
Technological innovation	Rapid	Slow	Rapid
Energy sector	Balanced use	Adaptation to local resources	Smooth transition to renewable
Environmental protection	Active management	Local and regional focus	Protection of biodiversity

*Population and per capita income projections are from [9].

It is inappropriate to assume that a simple extrapolation of historical and current trends would precisely represent the future landscape, as landscape trends change in response to socioeconomic (and climate) conditions. By using a multiple scenario approach, we could capture a range of potential future landscapes under different socioeconomic assumptions. Each of the three scenarios (see Table 1) captures different levels of clear-cutting, with the environmentally focused B1 scenario representing lower levels of clear-cutting than the economically focused A1B and A2 scenarios. In FORE-SCE, no clear-cutting was allowed to occur within National Park or Wilderness Area lands, and rural development in each of the scenarios was defined to be extremely low.

The final product was 2006–2050 annual land cover and land use maps at 250-m resolution, containing the 17 classes of Open water, Developed, Mechanical disturbed national forest, Mechanical disturbed other public forest, Mechanical disturbed private lands, Mining, Barren, Deciduous forest, Evergreen forest, Mixed forest, Grassland, Shrubland, Cultivated crop, Hay/pasture, Herbaceous wetland, Woody wetland, and Perennial snow/ice [39]. When a land was converted from one type to another (e.g., forest conversion to grassland), the carbon change was quantified based on IPCC good practice guidance [40]. Therefore, carbon removal due to forest clear-cutting could be tracked.

#### Wildfire projection from 2006 to 2050

Wildfire projections, driven by daily weather conditions, were generated for the study area using a spatially explicit simulation model [41]. Daily weather data were generated by temporally disaggregating the projected monthly temperature and precipitation data [42] and historical daily weather data with 1/8° spatial resolution [43]. Wind direction and speed information was provided by the North American Regional Reanalysis [44]. The Mountain Climate Simulator (MT–CLIM) [45] was used to calculate relative humidity using the daily temperature and precipitation data. Daily live and dead fuel moistures, and wildland-fire behavior indices were then estimated using the National Fire Danger Rating System (NFDRS) algorithms [46].

Wildfire ignition locations were stochastically generated using General Linear Models (GLMs) and fit using historical weather data and fires. The spread of wildfires from individual ignition locations was simulated with the Minimum Travel Time (MTT) algorithm [47] using surface and canopy fuels [48], topography (elevation, slope, and aspect), weather (wind speed and direction), and live and dead fuel moisture data. The outputs produced by the MTT algorithm included the burned pixels as well as metrics of crown fire activity, which were used as a proxy measure of burn severity (low, medium, and high).

For each pixel burned in the simulations, the First Order Fire Effects Model (FOFEM) [49] used fuel loads along with fuel moistures to estimate the amount of forest litter and downed deadwood consumed. The consumption of duff (decaying forest litter), trees, plants, and shrubs was estimated as a function of the region, season, fuel moistures, and fuel loads. When calculating emissions with the FOFEM, 20-, 60-, and 100-percent canopy consumption was assumed for low, moderate, and high burn severity, respectively, on the basis of published literature [50]. Therefore, carbon removal by fire consumption was quantified.

Before wildfire projections were made, the ignition and spread components of the wildland-fire modeling system were calibrated with the historical Monitoring Trends in Burn Severity (MTBS) data [51]. More details of the wildfire modeling methodology can be found in Hawbaker, et al. [41,52].

#### Biogeochemical cycles and carbon modeling

The GEMS modeling system was used in this study. GEMS was designed to provide spatially explicit biogeochemical model simulations over large areas. It was developed to better integrate well-established ecosystem biogeochemical models and employs a Monte-Carlo–based ensemble approach to evaluate model uncertainties [18]. The underlying biogeochemical model is CENTURY 4.0 (http://www.nrel.colostate.edu/projects/century, see Metherell, et al. [53]). The details of handling data discrepancy (e.g., when comparing MODIS NPP with modeling NPP, the former could capture real-time disturbances, but the latter did not incorporate these disturbances) and data frequency (e.g., low FIA measurements probably did not capture tree mortality sufficiently) during the model initialization, calibration, and validation can be found in Liu, et al. [18].

For model initialization, soil thickness, organic carbon storage, texture (fractions of sand, silt, and clay), bulk density, and drainage were initialized from the soil database. The total soil organic carbon pool was partitioned into active (5 percent), slow (45 percent), and passive (55 percent) pools. These percentages were only used for starting initialization. The model used 10–20 years to approach soil carbon equilibrium and the values were close to SSURGO soil carbon. Forest biomass carbon pools, including the forest litter biomass, aboveground live biomass, belowground live biomass, down deadwood biomass, and standing dead biomass, were derived from the data of Forest Inventory and Analysis (FIA, http://www.fia.fs.fed.us/), forest type (evergreen, broadleaf, and mixed), and the forest age-carbon stock relation.

For model calibration, the 2001–2005 observed data were compared to modeling output and the model parameters were adjusted to minimize the difference between simulations and observations. The observed data for calibration included (1) county-based grain-yield-survey data by crop type, published by the U.S. Department of Agriculture (USDA) [33]; and (2) 250-m MODIS Net Primary Production (NPP) for forests and grasslands. During the calibration process, the potential maximum production parameter (PRDX) was adjusted to minimize the grain yield difference (i.e., modeled grain yield versus USDA county-level grain yield) and the forest NPP difference (i.e., modeled NPP versus MODIS NPP at the county level).

For model processing, CENTURY simulated NPP, photosynthetic allocation, litter fall, mortality, decomposition of plant tissues, and soil organic carbon at monthly steps from 2006 to 2050. The annual CO_2_ concentration increase was included in the modeling, but CO_2_ concentration remained constant in the spatial domain. Important monthly and annual carbon-related variables were output from the nine modeling combinations:

- FSYSC: Total ecosystem carbon storage
- FRSTC: Total living carbon, including both aboveground and belowground biomass
- SOMSC: Total soil carbon excluding litter and structural carbon
- CPRODA: Net carbon production (i.e., NPP)
- TCREM: Carbon removal from ecosystems by clear-cutting and fire consumption

The modeling performance was validated by comparing the simulation with the corresponding observation, which included USDA forest biomass values, aboveground biomass from the National Biomass and Carbon Dataset 2000 [54], MODIS NPP, and the USDA grain yield for 2006, 2008, and 2010.

For the nine simulation experiments, the variation of carbon outputs is expressed as the “standard deviation (V)” defined as

1 $$ V = \sqrt{\frac{1}{N}{\displaystyle \sum_{i=1}^N}{\left({x}_i-\mu \right)}^2} $$

where N is the number of the data-scenario combinations of 9, x_i_ is each individual variable, and μ is the mean of x_i_.

To compare the different effects of fires on carbon modeling, the forest area regenerating from the 1988 fires (age is 18 years in 2006) was selected as young forest and a neighboring unburned forest stand was selected as mature forest (see Figure 1).

## Results

### Carbon pools and fluxes for mature and young forest in 2006

For the selected mature and young forests (see Figure 1), different combinations of data and scenarios in our modeling resulted in the carbon storage and fluxes depicted in 2006 (Table 2). When averaged over the data-scenario combinations, the young and mature forest respectively had 7,874 gC/m^2^ and 9,534 gC/m^2^ for the total ecosystem carbon, 3,389 gC/m^2^ and 4,310 gC/m^2^ for total living biomass, and 301 gC/m^2^ and 324 gC/m^2^ for net primary production (NPP). The standard deviations in each data-scenario implied there was substantial spatial variation in carbon storage and fluxes.

**Table 2:** **Modeling results for mature and young forest in 2006**

	**cgcm**						**Csiro**						**miroc**						**Data/scenario combination**
	**B1**		**A2**		**A1B**		**B1**		**A2**		**A1B**		**B1**		**A2**		**A1B**		
	**mean**	**std**	**mean**	**std**	**mean**	**std**	**mean**	**std**	**mean**	**std**	**mean**	**std**	**Mean**	**std**	**mean**	**std**	**mean**	**std**	**mean**
FSYSC																			
*Young*	7889	2751	7805	2726	7885	2746	7898	2760	7878	2750	7874	2747	7868	2747	7853	2739	7913	2760	7874
*Mature*	9551	1589	9428	1554	9498	1564	9575	1600	9524	1581	9622	1633	9510	1577	9478	1561	9619	1616	9534
FRSTC																			
*Young*	3403	1398	3343	1378	3374	1393	3405	1399	3392	1396	3406	1389	3390	1396	3369	1389	3418	1405	3389
*Mature*	4322	1054	4251	1038	4274	1043	4339	1058	4300	1048	4364	1070	4300	1048	4272	1042	4364	1065	4310
CROPDA																			
*Young*	322	69	228	66	282	63	331	74	302	66	318	128	303	62	273	59	348	81	301
*Mature*	338	58	239	40	274	50	365	66	306	57	410	98	309	52	269	51	405	83	324

• Note: FSYSC refers to total ecosystem carbon storage (gC/m^2^); FRSTC refers to total living carbon, including both aboveground and belowground biomass (gC/m^2^); and CPRODA refers to net carbon production (gC/m^2^/year).

### Carbon change for entire GYE

**Figure 5:**
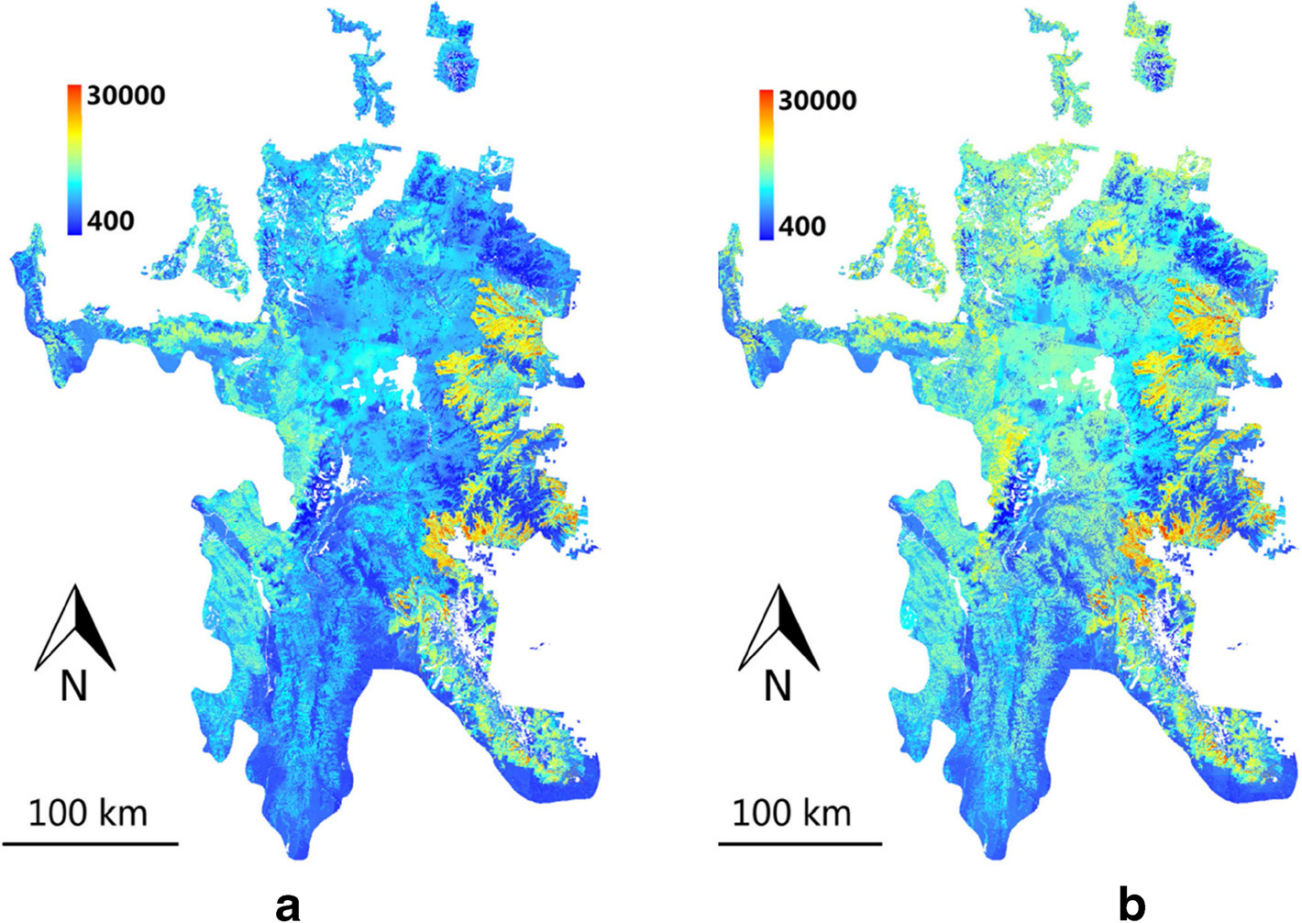
**Total ecosystem carbon (gC/m** ^**2**^ **) in (a) 2006 and (b) 2050 resulted from CGCM-A2.** Note the abrupt change in the eastern part was caused by the difference between SSURGO (right side) and STATSGO (left side). The mean difference of soil organic matter between SSURGO and STATSGO along this abrupt change line was about 6,993 gC/m^2^.

We produced spatially explicit layers for variables of total ecosystem carbon, total living carbon, total soil carbon, net carbon production, and carbon removal from 2006 to 2050 at 250-m resolution. Maps for all nine modeling combinations were produced, but only the total ecosystem carbon data in 2006 and 2050 for CGCM-A2 are shown in Figure 5 as a demonstration. Figure 5 shows there are significant spatial variations in total ecosystem carbon, indicating the heterogeneity of carbon storage. In Figure 5a, the mean of total ecosystem carbon in 2006 was 7,885 gC/m^2^ and the standard deviation was 4,688 gC/m^2^. In Figure 5b, the mean of total ecosystem carbon in 2050 was 10,076 gC/m^2^ and the standard deviation was 5,325 gC/m^2^. This phenomenon of landscape heterogeneity could be observed for all map layers.

**Figure 6:**
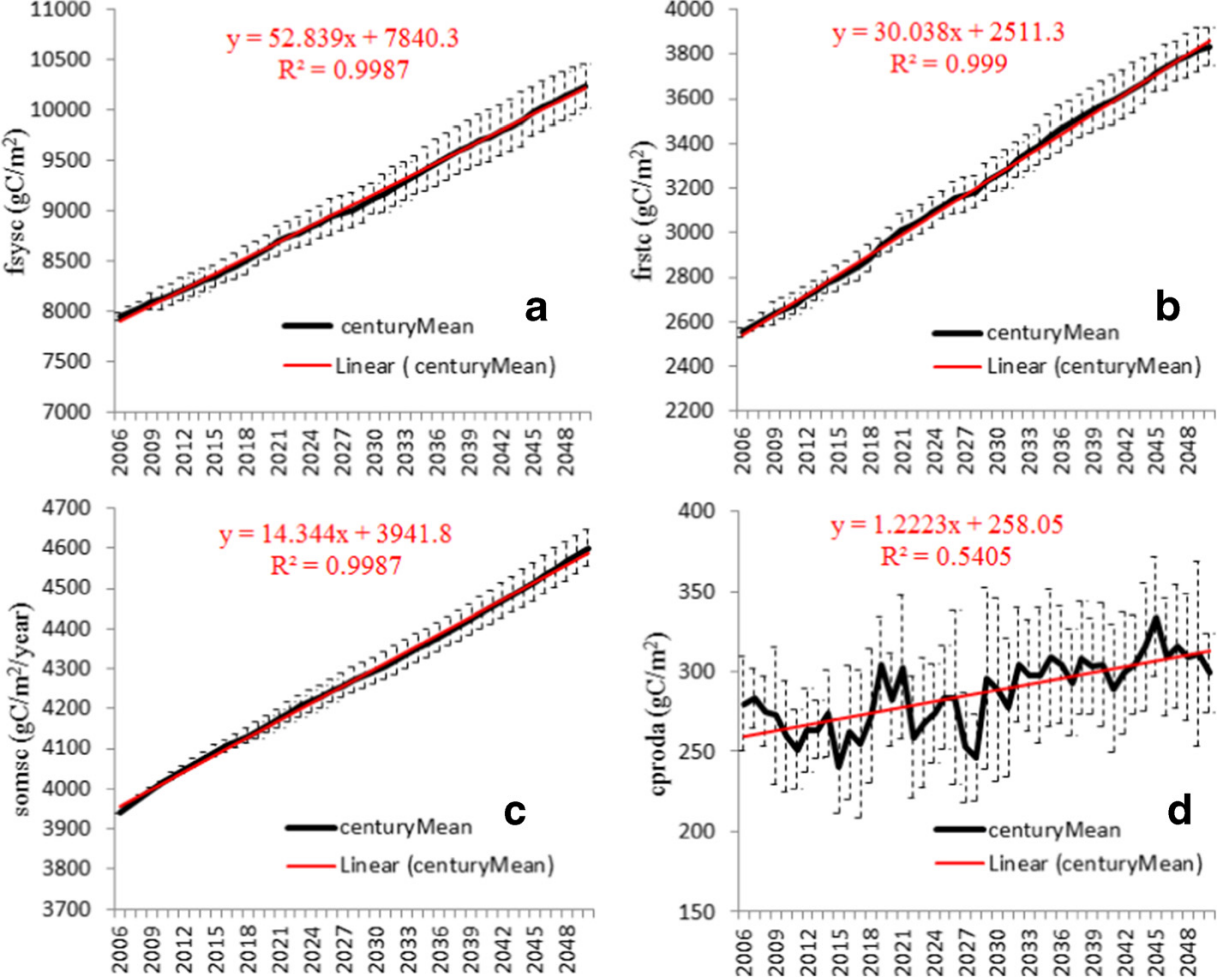
**Modeling results of total ecosystem carbon (FSYSC) (a), live biomass carbon (FRSTC) (b), soil organic carbon (SOMSC) (c), and net primary production (CPRODA) (d) in Greater Yellowstone Ecosystem.** Red lines are linear regressions, and the error bars are standard deviations calculated with equation 1.

Our modeling also produced time series carbon pools and fluxes (Figure 6). In the GYE ecosystem, all nine data-scenario combinations run under the CENTURY model showed increasing trends of total ecosystem carbon, indicating incrementally more carbon will be sequestered in the GYE ecosystem. This implied GYE will be a carbon sink in a future with climate change. The variance among the combinations was within 5.5%, indicating the difference was very little. Therefore, we calculated the annual mean of total ecosystem carbon with an error bar of standard deviation and performed a linear regression (Figure 6a), which shows the total ecosystem carbon increased steadily from 7,942 gC/m^2^ in 2006 to approximately 10,234 gC/m^2^ in 2050 (i.e., 28.9% increase), with a mean value of 9,056 gC/m^2^. The extra carbon sequestration was 2,292 gC/m^2^ during 2006–2050, and the average annual increasing rate, which was reflected by the slope, was 53 gC/m^2^/year.

Total live carbon is an important component of total ecosystem carbon. The annual change of this carbon pool (Figure 6b) increased steadily from 2,551 gC/m^2^ in 2006 to 3,833 gC/m^2^ in 2050. The average annual rate increase is about 30 gC/m^2^/year, which accounts for 56.6% of the annual rate increase of 53 gC/m^2^/year in total ecosystem carbon. The time-series total soil carbon (Figure 6c) also steadily increased from 3,939 gC/m^2^ in 2006 to 4,601 gC/m^2^ in 2050. The average annual rate increase was 14.3 gC/m^2^/year, which contributed 27% of the annual rate increase of 53 gC/m^2^/year of total ecosystem carbon. All of these changes in total ecosystem carbon, total live carbon, and total soil carbon indicated the GYE under climate change is a carbon sink and can sequester 2,292 gC/m^2^, and 83.6% of the extra carbon sequestrated during 2006–2050 is attributed to the carbon pools of live biomass and soil organic matter.

Associated with the increased total soil carbon, live biomass carbon, and total ecosystem carbon, forest productivity increased as reflected by the net carbon production (Figure 6d). Different data-scenario combinations resulted in significant differences, which could be reflected in the high standard deviations ranging from 18.3 gC/m^2^/year to 57.7 gC/m^2^/year during 2006–2050, indicating GCMs have great effect on NPP. However, linear regression shows net carbon production increased slightly from 260 gC/m^2^/year in 2006 to 310 gC/m^2^/year in 2050 with an annual rate increase of 1.22 gC/m^2^/year, implying the forest productivity increased approximately 19.2% under climate change.

### Forest clear-cutting, wildfires, and carbon removal

**Figure 7:**
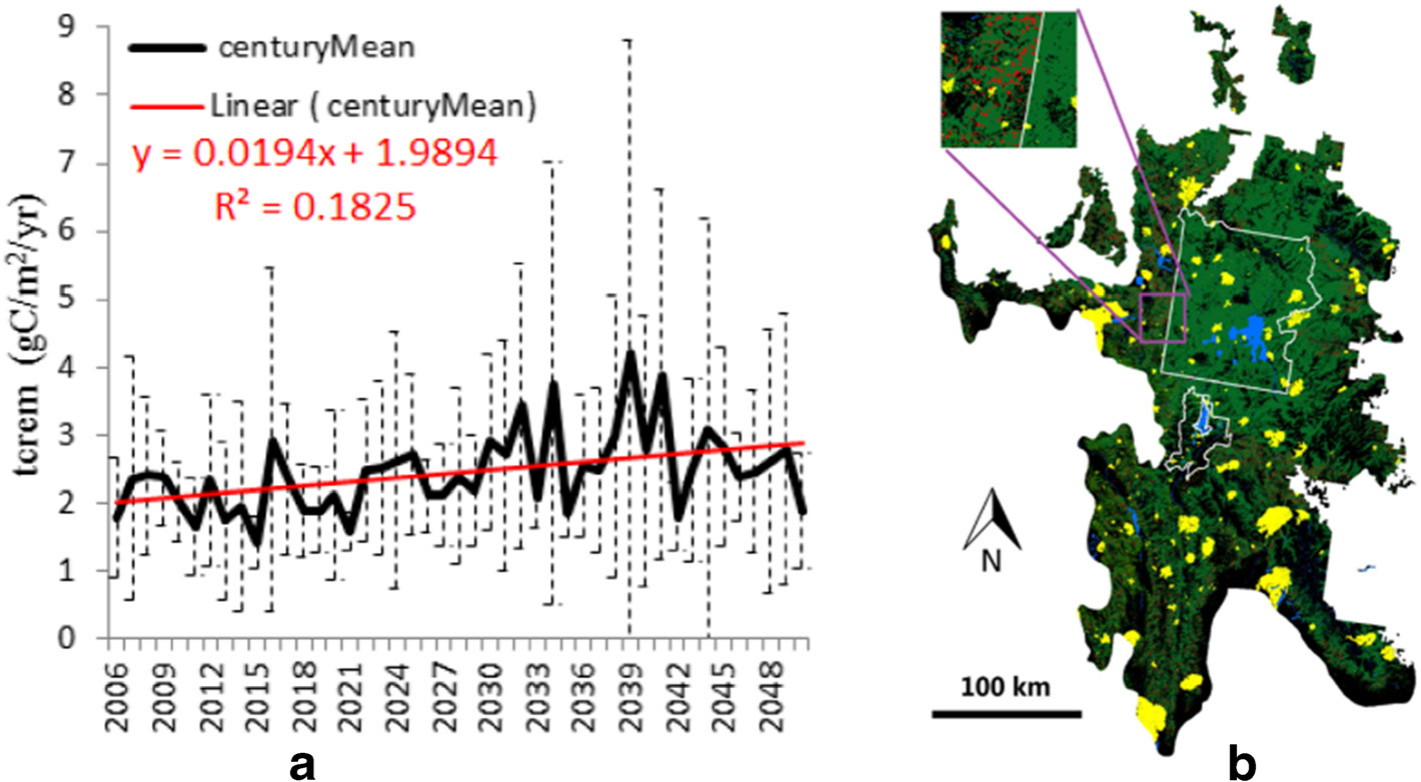
**Carbon removal from simulated forest clear-cutting and wildfires (i.e., TCREM) in GYE (a) and the wildland fires (yellow dots) and forest clear-cutting (red dots) from 2006 to 2050 (b).** Blue areas are water bodies, white lines are the national park borders, and green areas are forest in 2006. Wildfires are modeled from the CGCM-A2 scenario and forest clear-cutting is modeled from the A2 scenario. Modeling results of other scenarios can be found in Tables 3 and 4.

Despite the increased net carbon gain and forest productivity during 2006–2050, forest clear-cutting and fires resulted in direct carbon removal (Figure 7). The standard deviation bars show this annual carbon loss from the removal events had high variation among scenarios and the magnitude ranged from 1.4 gC/m^2^/year in 2015 to 4.2 gC/m^2^/year in 2039, with a mean removal of about 2.44 gC/m^2^/year during 2006–2050 (Figure 7a). The linear slope of 0.02 indicates there is a very low annual rate increase of carbon removal from clear-cutting and wildfires. The total carbon removal during 2006–2050 was 109.6 gC/m^2^, which accounts for only 1.2% of the mean ecosystem carbon level of 9,056 gC/m^2^ over 45 years, and thus is negligible.

Clear-cutting and fires have different distribution in national parks (NP) and national forests (NF) (Figure 7b). Our modeling indicates wildfires would occur across the entire GYE. However, the clear-cutting was only distributed in NF, which can be clearly distinguished by the abrupt change along the border between NP and NF (see the magnified portion in Figure 7b). The clear-cutting ratio in NF ranged from 4.87% under the B1 scenario to 6.34% under the A1B scenario with an average ratio of 5.64% (Table 3). In addition, Table 4 shows the burned forest ratio in NP ranged from 3.19% under the CSIRO-B1 scenario to 12.25% under CSIRO-A1B with an average ratio of 6.08%. Table 4 also shows 5.72%–7.49% (mean is 6.55%) of the forested area in NF would be burned under future climate change. With clear-cutting and wildfires combined, 6.11% of the forested area in NP and 12.19% of the forested area in NF would be affected. Together, the area of clear-cutting and wildfires in GYE would account for 10.87% of total forested area during 2006–2050.

**Table 3:** **The area of forest cutting within national forests under climate scenarios**

	**National forest**		
	**B1**	**A2**	**A1B**
Clear-cutting area (km^2^)	1288	1510	1677
Clear-cutting ratio (%)*	4.87	5.71	6.34

*In 2006, forested area within NP is 7,321 km^2^ and forested area within NF is 26,457 km^2^.

**Table 4:** **The area of forest fires within national parks and national forests under climate change scenarios during 2006–2050**

	**National park**									**National forest**								
	**cgcm**			**csiro**			**miroc**			**cgcm**			**csiro**			**miroc**		
	**B1**	**A2**	**A1B**	**B1**	**A2**	**A1B**	**B1**	**A2**	**A1B**	**B1**	**A2**	**A1B**	**B1**	**A2**	**A1B**	**B1**	**A2**	**A1B**
Burned forest (km^2^)	344	322	441	233	275	897	706	510	280	1837	1607	1664	1515	1821	1981	1810	1689	1675
Burned forest (%)*	4.70	4.40	6.02	3.19	3.75	12.25	9.65	6.97	3.82	6.94	6.07	6.29	5.72	6.88	7.49	6.84	6.38	6.33

*In 2006, forested area within NP is 7,321 km^2^ and forested area within NF is 26,457 km^2^.

It should be emphasized that within national parks, fires were modeled, but clear-cutting was not allowed. Any increased forest disturbance in national park land would be solely due to a more active fire regime. For forest disturbance related to clear-cutting outside of national park lands and national forest lands, the use of multiple scenarios allows us to examine multiple socioeconomic pathways affecting forest management in the region. We believe it is more insightful to provide a range of potential futures than to model what will “probably” happen. The B1 scenario maintained levels of clear-cutting similar to 2005. The other two scenarios projected increases due to the socioeconomic assumptions within those scenarios.

## Discussion

GCM outputs are the basis for carbon projection under climate change. Our precipitation change ranged from −0.387 mm/y to 1.916 mm/y (Figure 4). In the study of Smithwick, et al. [14], the precipitation was predicted to increase 21 mm [from Hadley (HAD) source] to 32 mm [from Canadian Climate Center (CCC) source] during 1994–2100 (i.e., 0.198 mm/y from HAD and 0.302 mm/y from CCC). This change rate was higher than found in this study. In Smithwick, et al. [14], the average annual maximum temperatures were expected to increase 2.8°C (HAD) to 4.3°C (CCC) (i.e., 0.026°C/y and 0.041°C/y), which falls within or is greater than the upper limit of our projection data (Figure 2). However, the average annual minimum temperatures used by Smithwick, et al. [14] were expected to increase 4.7°C (HAD) to 9.1°C (CCC) (i.e., 0.044°C/y and 0.086°C/y), which falls within our projection data (Figure 3). The difference may be attributed to at least two reasons. First, precipitation prediction is more difficult than temperature prediction, which may lead to greater variance among GCMs. Second, the time scale of their projection was 1994–2100, which was longer than our time scale of 2006–2050. The variation among different GCMs indicated a more robust data source to be used for future carbon modeling.

Wildfires result in forest age mosaics that affect carbon storage. Our NPP values for 18-year-old forest stands and mature forest were 301 gC/m^2^ and 324 gC/m^2^, respectively; these values are comparable to results from previous studies. Litton, et al. [55] examined how aboveground NPP (ANPP) and belowground NPP (BNPP) varied with fire-initiated differences in tree density and stand age in lodgepole pine stands in Yellowstone National Park. They found the annual ANPPs were 59, 122, 156, and 218 gC/m^2^ and the annual BNPPs were 68, 237, 306, and 382 gC/m^2^ for low-, moderate-, and high-density young stands (13-year-old) and mature stands, respectively. Their values indicate the annual NPP, which is the addition of ANPP and BNPP, was 316 gC/m^2^ for 13-year-old forest stands and 600 gC/m^2^ for mature forest stands. Our NPP for 18-year-old forest stands was 301 gC/m^2^, which was lower than the measured NPP value of 316 gC/m^2^ by Litton, et al. [55] but higher than the modeled 2004 NPP of 245–253 gC/m^2^ for early and middle successional lodgepole pine stands by Crabtree, et al. [56]. Our NPP for mature forest was 324 gC/m^2^, which was 46% lower than the value of 600 gC/m^2^ by Litton, et al. [55] but 28 ~ 35% higher than the 2004 NPP value of 240–253 gC/m^2^ for middle and late successional lodgepole pine stands reported by Crabtree, et al. [56]. In general, both our young and mature forest NPP were lower than the measured NPP of Litton, et al. [55]. One possible reason is that these stands were located in the areas where STATSGO soil data were used (see Figure 5). The nutrition supplies, which affect the plant production, were lower in the coarser STATSGO database than in SSURGO.

In our study, the total living carbon of young forest in 2006 (regenerating from 1988 fires) was 3,389 gC/m^2^. Kashian, et al. [57] measured carbon pools for 77 lodgepole pine stands in and around Yellowstone National Park (YNP) along a 300-year chronosequence. They showed the live vegetation carbon can be modeled from stand age using a Michaelis-Menton function. According to this function, the forest burned in 1988 (with an age of 18 in 2006) is 3,417 gC/m^2^. Our result of 3,389 gC/m^2^ agreed with the prediction of 3,417 gC/m^2^ very well. However, the mature forest (with an age of 100–300) predicted by the expression of Kashian, et al. [57] is 7,775–9,560 gC/m^2^, which is higher than our result of 4,310 gC/m^2^. Nevertheless, Kashian, et al. [57] also showed their total living biomass carbon had significant variation among mature forest stands, and our value of 4,310 gC/m^2^ still fell within their range.

Our results showed the young forest in 2006 had a total ecosystem carbon of 7,874 gC/m^2^ and the mature forests have a total ecosystem carbon of 9,534 gC/m^2^. Kashian, et al. [57] found the total ecosystem carbon increased with stand age following a Michaelis-Menton function. Using this function, the 1988 burned forest (with an age of 18 in 2006) would have a mean total ecosystem carbon of 10,328 gC/m^2^, which is 31% higher than the value determined for our study. Nevertheless, they also showed the measured total ecosystem carbon for young forest ranged from 6,000 to 15,600 gC/m^2^. In our study, as depicted in Figure 5, the soil carbon represented by STATSGO, which was used in our young forest area, was 6,993 gC/m^2^ lower than that of SSURGO, which represents closer to actual conditions. If the bias of 6,993 gC/m^2^ was simply added, our result would be 14,867 gC/m^2^, which falls within the range of 6,000 to 15,600 gC/m^2^ of Kashian, et al. [57]. Similarly, using their function, the mature forest (assuming an age of 100–300 years) would have a total ecosystem carbon of 14,815 to 16,745 gC/m^2^, which is higher than our value of 9,534 gC/m^2^. When the bias of 6,993 gC/m^2^ was simply applied, our total ecosystem carbon for mature forest was 16,527 gC/m^2^, which was close to the 14,815–16,745 gC/m^2^ predicted by Kashian, et al. [57]. The value of 16,527 gC/m^2^ was also close to the 17,079 gC/m^2^ measured by Litton, et al. [55] and 17,900 gC/m^2^ modeled by Smithwick, et al. [15] for mature lodgepole pine stands.

Whether an ecosystem is a carbon sink or source is important for climate mitigation. We found GYE will be a carbon sink under climate change; this finding agrees with Smithwick, et al. [14], who also found the increasing carbon sequestration and suggested the potential for an increase in net carbon storage in GYE lodgepole pine forests under projected future climates. We found the forest productivity increased approximately 19.2% under climate change. This finding coincides with Melillo, et al. [58], who found that temperate ecosystem net primary productivity increased under climate change due to the effect of elevated temperature in enhancing the mineralization of nitrogen in the soils. The elevated temperature was also observed in our study, as shown in Figures 2 and 3. Our general increasing trend in forest productivity is consistent with Smithwick, et al. [14], who revealed the same trend, but our magnitude of 19.2% is lower than their estimates of 25% (from HAD) and 36% (from CCC). The reasons for the difference came from the different climate data sources, but this was also caused by the different time scale (our 2006–2050 versus their 1994–2100): for many climate data sets, temperature and precipitation change much more after 2050 than before 2050.

GCMs have uncertainties and can influence carbon modeling. We found the variance of the total ecosystem carbon among the data-scenario combinations was within 5.5%, indicating the difference was very little. This finding is in contrast to Morales, et al. [59], who showed the choice of the GCM strongly influenced carbon balance in Europe. However, this finding agrees with Smithwick, et al. [15], who showed total ecosystem carbon stocks in GYE varied little (<10 percent) among future climate scenarios for a given A2 emission and fire-event pathway. Clearly, geography is attributed to this variation, indicating different landscapes have different carbon sensitivity to climate change.

The changing climate, wildfires, land use, and biogeochemical processes (e.g., forest productivity) comprehensively alter the carbon sequestration in a spatially heterogeneous manner. Understanding how concurrent changes in climate, disturbance regimes, and land use affect carbon storage in a spatially explicit manner and the accompanying uncertainties are critical but also challenging. Our method combined the concurrent changes in climate, fire, and land use for carbon modeling from 2006 to 2050 at 250 m resolution. We could also quantify the uncertainties from GCMs by processing the same CENTURY model with data from different sources. The result can help GYE stakeholders manage carbon sequestration as an important ecosystem service, and the methodology developed in this study can be applied to other regions to reveal spatiotemporal carbon dynamics under climate change. However, there are several potential areas of improvement from our current approach.

First, bark beetles (*Curculionidae: Scolytinae*) are a major native disturbance agent in most temperate coniferous forests. Since 1999, a warming climate in the Northern Rockies has coincided with beetle eruptions, which have exceeded historical records of the previous 125 years [60,61]. The outbreak of bark beetles can influence more land area than wildfires and result in a change in structure, function, and composition of forest ecosystems [19], and the impact could convert the forest from a small net carbon sink to a large net carbon source both during and immediately after the outbreak [62,63]. Beetle outbreaks are occurring throughout the entire distribution of the GYE [64]. Climate change has contributed to the unprecedented extent and severity of this outbreak [62]. Given the current mortality caused by bark beetles and projections for the future, this disturbance would shift the balance toward reduced photosynthesis capability and greater forest floor and soil carbon accumulation due to overstory tree mortality and subsequent coarse woody debris formation. If this disturbance is considered, GYE may even shift to a carbon source. Therefore, future carbon modeling needs improvement in considering bark beetle infestation.

Second, Romme, et al. [23] projected the probable effects on several representative species and community types in the GYE and found the extent of alpine vegetation in the ecosystem decreased in all scenarios. Bartlein, et al. [65] also projected the biotic response to future climate changes in Yellowstone and found the range of high-elevation species decreases and some species become regionally extirpated. The species redistribution affects ecosystem carbon sequestration, but given the time span modeled of 44 years (2006 to 2050), broad-scale change in vegetation communities may have been unlikely. For a longer time span, such as 100 years, vegetation shifting may be necessarily taken into account.

Third, after fire, net carbon loss to the atmosphere can persist for over a century [66], and fire intervals in coniferous forest are often more than 100 years [5]. This indicates that understanding the carbon cycle of a full fire cycle requires a time scale beyond 100 years. The period of our study was from 2006 to 2050, which is much shorter than this time scale.

Finally, the modeling system used in this study was originally developed for carbon analysis at the national scale. When the national-scale system was applied to the local study such as GYE, some issues can arise and there is a potential to reduce the uncertainty. For example, the models were not fine-tuned specifically for GYE, and the previous fire events such as the big fire of 1988 were not adequately represented in model simulation. Furthermore, both STATSGO and SSURGO soil databases were used in our modeling. Because the STATSGO database has less detailed information than the SSURGO database, the soil organic matter and nutrient supplies differ, which affects the simulation of ecological processes such as net primary production and soil organic decomposition. It is necessary to refine the soil data such as Yellowstone soil database [67] to improve the modeling results.

## Conclusion

The GYE is a temperate ecosystem that, based on the assumptions behind climate projections, will likely be subject to an elevated temperature, but change in precipitation in future decades varies among GCMs. With the changing climate, wildfires, land use, and the processes regulating the carbon cycle will be changed simultaneously. The concurrent change will lead to increasing total ecosystem carbon from 7,942 gC/m^2^ in 2006 to approximately 10,234 gC/m^2^ in 2050 with an annual rate increase of 53 gC/m^2^/year. This finding indicates climate change can enhance the carbon sink characteristics of the GYE.

With an elevating temperature, the NPP will increase approximately 19.2%. Total live biomass carbon will increase from 2,551 gC/m^2^ in 2006 to 3,833 gC/m^2^ in 2050 with an annual rate increase of 30 gC/m^2^/year. Soil organic matter will increase from 3,939 gC/m^2^ in 2006 to 4,601 gC/m^2^ in 2050 with an annual rate increase of 14.3 gC/m^2^/year. These two carbon pools explained 56.6% and 27% of the extra carbon sequestration, respectively. This finding indicates the capability of vegetation is almost double that of soil in potential for sequestering extra carbon.

Clear-cutting and fires have different distributions in national parks (NP) and national forests (NF), with fires occurring in both NF and NP but clear-cutting mainly occurring in NF. In NF, 5.64% of the forest will be cut and 6.55% will be burned during 2006–2050. In NP, 6.08% of the forest will be burned during 2006–2050. Together, clear-cutting and wildfires in GYE will affect 10.87% of total forested area during 2006–2050. This finding indicates clear-cutting and wildfires under climate change may have great effect on this ecosystem, although the direct carbon removal by these events is insignificant.
